# Breast Cancer Survivorship and Level of Institutional Involvement Utilizing Integrative Oncology

**DOI:** 10.1155/2021/4746712

**Published:** 2021-12-18

**Authors:** Terri Crudup, Linna Li, Jennifer Wright Dorr, Elizabeth Lawson, Rachel Stout, Pedram Vazifeh Niknam, Judi Jones, Robert G. Steen, Susan Casner, Lynn L. Lu, Yi Wang, James Scott, Shelby Zanine, Stacey Robertshaw, Gabriella Broderick, Simarpreet Singh, Jingsong Lu, Li Zhou, Vaishali Palommella, Tye Harris, Michael Hanamirian, Mula Shivani Reddy, Bruce Cowgill, Jen Rice, Avinash Nagaraja, Wayne Jonas

**Affiliations:** ^1^Primary Intelligence, IQVIA, Plymouth Meeting, PA, USA; ^2^Radiation Oncology, Bryn Mawr Hospital, Bryn Mawr, PA, USA; ^3^Integrative Health Programs, Samueli Foundation, Alexandria, VA, USA; ^4^Real World Oncology, IQVIA, Plymouth Meeting, PA, USA; ^5^Advanced Analytics, IQVIA, Plymouth Meeting, PA, USA; ^6^Primary Intelligence, IQVIA, Gurgaon, Haryana, India; ^7^Primary Intelligence, IQVIA, Bengaluru, Karnataka, India

## Abstract

**Objective:**

Integrative oncology is widely used by patients with breast cancer. This study aims to investigate the relationship between the survival outcomes of breast cancer patients and the level of involvement in integrative oncology at the institutions treating them.

**Methods:**

Claims-based data were used to find 4,815 newly diagnosed breast cancer patients treated between January 2013 and December 2014 for survival analysis. A scoring system was developed by asking oncologists about their institutions' efforts to educate, support, and provide funding for 12 complementary and lifestyle approaches. Cohort analysis using two-tailed chi-square and a separate multivariate model using SMOTE and lasso regression were used. Nine variables across patient and institutional profiles were included. The model coefficients were exponentiated and presented as odds ratios.

**Results:**

173 patients mapped to 103 institutions and 103 oncologists. The median patient age was 51, and 8% were metastatic. Institutions were scored for integrative oncology involvement and placed into four cohorts. Low-scoring institutions showed less effort to educate, support, and provide integrative therapies compared to others. The 5-year survival of patients in the low cohort was directionally but not significantly lower than others. In the multivariate model, a composite integrative oncology score was shown to increase 5-year survival odds three times for institutions in the low-mid cohort and 48% in the mid-high, compared to the low.

**Conclusion:**

Crossing the threshold beyond ‘low' involvement in integrative oncology represents a new path to incremental survival benefit for many cancer patients. Entities invested in the survival of breast cancer patients should increase education, access, and funding for a core set of six therapies: nutrition counselling, exercise counselling, patient support groups, spiritual services, meditation, and psycho-oncology support.

## 1. Introduction

Cancer impacts the whole person affecting all dimensions of the individual—mind, body, and spirit. Care for the cancer patient is correspondingly complex and increasingly individualized. While the oncology community relies on conventional medicine as the backbone of therapy, many patients combine complementary and lifestyle therapies in an approach known as integrative oncology. Integrative oncology is defined as “a patient-centered, evidence-informed field of cancer care that utilizes mind-body practices, natural products, and/or lifestyle modifications from different traditions alongside conventional cancer treatments” [1]. Integrative approaches encompass numerous modalities including patient support groups, massage, and nutritional and exercise counselling. While well-known and often recommended, they have variable availability in cancer treatment. There is a growing body of evidence that adding complementary and lifestyle approaches to conventional oncology treatment benefits patients by helping them manage the side effects of treatment [2], improving patient-reported outcomes [3], and contributing to improved overall survival [4, 5]. This study aimed to investigate the relationship between institutional involvement in integrative oncology and survival in breast cancer patients.

## 2. Materials and Methods

This study used data from US breast cancer patient claims, paired with a survey among 103 US oncologists, to investigate the relationship between 5-year survival outcomes of breast cancer patients and the level of education, support, and funding across 12 complementary and lifestyle therapies in the institutions treating those patients.

### 2.1. Claims Data and Survey Analytics

The IQVIA PharMetrics® Plus database was used to meet the study objectives. PharMetrics Plus includes longitudinal, adjudicated administrative claims for more than 190 million unique health plan members across the United States [6]. Data include inpatient and outpatient diagnoses and procedures, retail and mail order prescription records, pharmacy and medical benefit information, inpatient stay and provider details, demographic variables, product type, payer type, health plan enrolment dates, and payments.

**Figure 1:**
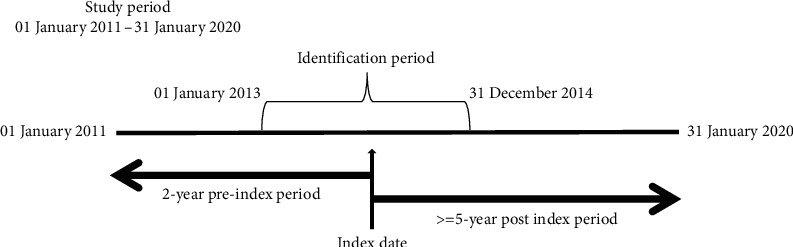
Breast cancer patients claim data study timeframes. The full study period is from January 01, 2011, through January 31, 2020. The identification period for breast cancer diagnosis was between January 01, 2013, and December 31, 2014. A 2-year preindex period (from January 01, 2011, through the index date) was used to apply exclusion criteria. A 5-year postindex period (from the index date through December 31, 2019) was used to assess survival.

Patients diagnosed with breast cancer between January 01, 2013, and December 31, 2014 (identification period) were selected (Figure 1). Codes from the international classification of diseases (both ICD-9 and ICD-10) were used to identify breast cancer diagnosis at any stage. Patients were required to have at least one confirmatory inpatient claim, or two confirmatory outpatient claims for breast cancer on different days during the identification period. The first claim with the diagnosis code was termed the index date.

The following exclusion criteria were applied: (1) age 17 or younger at the index date; (2) did not have two years of continuous enrolment before the index date; (3) had a breast cancer claim or diagnosis in the two-year preindex period (“newly diagnosed” only); (4) were high-risk based on a diagnosis of HIV (human immunodeficiency virus) or secondary cancer; and (5) did not have five years of continuous enrolment following the index date.

The PharMetrics Plus database lacks a variable for mortality, a discharge status of “expired,” and does not detect mortality in the community. Instead, a previously established claims death proxy method used by Joyce et al. and Pelletier et al. was applied to identify and quantify mortality. This method used a qualifying medical event and the absence of claims within 180 days following the event date to signify patient death [7, 8].

Additional profiling of the patients' conventional medical treatments was conducted using treatment and procedural codes from claims data.

Treating oncologists were defined as those with at least five medical claims from the same patient on different days during the postindex period. The treating oncologists were assigned to their “best affiliation” practice setting address using the IQVIA OneKey® database. The process to define “best affiliation” pulls physician physical addresses from several professional sources, weights each address to provide a confidence ranking, and then selects the professional location (healthcare organization) with the most evidence as the primary practice location.

Oncologists from the “best affiliation” institutions were invited to take part in a 20-minute survey about the integrative oncology involvement of their institutions in 2013–2014 to match the patient identification period. Screening criteria was required which are as follows: (1) currently a full-time practicing oncologist; (2) practicing at one of the institutions where the study pool patients were treated, both currently and in 2013–2014; (3) comfortable responding to questions about “complementary and lifestyle therapies offered or supported at their institution in 2013-2014”; and (4) currently treat at least 10 breast cancer patients in a typical 3-month period. Only one oncologist from each institution was allowed to take the survey.

There are currently no standard scoring systems for measuring level of involvement in integrative oncology. To construct a measurement system, IQVIA partnered with the Samueli Foundation, a leader in the field of integrative medicine. Following a targeted literature review on the use of complementary therapies by breast cancer patients, 12 therapies were chosen to be measured and relabelled as “complementary and lifestyle therapies” (see Results section).

By adapting the principles of awareness, interest, desire, and action in the consumer purchase funnel [9], each of these 12 therapies was measured across three constructs: educate, support, and provide. To measure how well institutions “educate” about the 12 therapies, questions were asked about efforts to increase patient awareness and knowledge (see Table 1). To measure “support,” questions were asked about efforts to offer or otherwise recommend across the 12 therapies. To measure “provide,” questions were asked about the institution covering the cost in part or in full of the 12 therapies. Each response was assigned points, as shown in Table 1, with higher points indicating stronger involvement. Finally, two additional questions were asked about the on-site oncology support staff and the efforts of the institution to handle patient questions about herbs and supplements. All questions were asked relative to the 2013–2014 timeframe to match the patient treatment period.

The composite integrative oncology involvement score was calculated by adding the total number of points based on responses to the five survey questions (shown in Table 1).

### 2.2. Statistical Analysis

The final set of patients for analysis was limited by the number of oncologists who completed the survey (see Results). The patient mortality marker was checked for the final breast cancer patient cohort and subsequent 5-year overall survival was calculated. To compare survival rates between cohorts with different levels of integrative oncology involvement, a chi-square test was used.

To fully analyse the impact of the integrative approaches, a multivariate model was constructed from the claims data with 5-year survival as the dependent variable. Due to the limited sample available to analyse, SMOTE and lasso regression were combined [10, 11]. SMOTE is a validated oversampling technique to adjust for low event rates (in our case, deceased), and lasso regression has been shown to include relevant variables when the number of variables is high relative to the sample size [12]. Numerous categorical independent variables were included. From patient claims data, the following are obtained: age at index date, US geographic region, metastatic status, and type of patient insurance health plan. From the survey data representing each institution, the following are obtained: composite integrative oncology score, NCCN (National Comprehensive Cancer Network) designation, NCI (National Cancer Institute) designation, ACO (Accountable Care Organization) affiliation, and practice setting. Each of these variables is present in the final output.

## 3. Results

### 3.1. Claims Data and Survey Analytics

Within the two-year identification period, 241,726 unique breast cancer patients were identified in PharMetrics Plus claims data using the definition of breast cancer diagnosis. After applying exclusion criteria, the study set of newly diagnosed adult breast cancer patients was 4,815. Among these, 475 (9.9%) were diagnosed as metastatic. Applying the mortality algorithm yielded a 5-year survival rate of 89.8%. After being mapped to treating institutions, a total of 2,758 oncologists representing 2,543 institutions were available for the survey research.

Of the 2,430 oncologists invited to take the survey, 675 attempted the screener (27.8% response rate). Of these, 401 did not meet the screener requirements, 96 did not complete the full survey, 61 were turned away due to the survey quota being filled, and two were removed after survey completion due to poor data quality. During the process of linking surveyed oncologists back to patients in the claims data, 12 oncologists were unable to be mapped using classification rules and were also removed. Therefore, a final sample of *n* = 103 oncologists representing 103 unique institutions was analysed. Most oncologists had between 1 to 2 patients in the claims data, producing a final study sample of 173 breast cancer patients.

The sample of 173 breast cancer patients had the following profile: the median age was 51 (range: 32–76), 14 of the patients (8%) were identified as metastatic, and 153 (88%) had a preferred provider organization (PPO) for plan type, compared to 20 (12%) with either a health maintenance organization (HMO), indemnity, or point of service. Using the definition of the census regions in the US, 15.6% resided in the Northeast, 40.5% resided in the South, 36.4% resided in the Midwest, and 7.5% resided in the West.

### 3.2. Statistical Analysis

**Figure 2:**
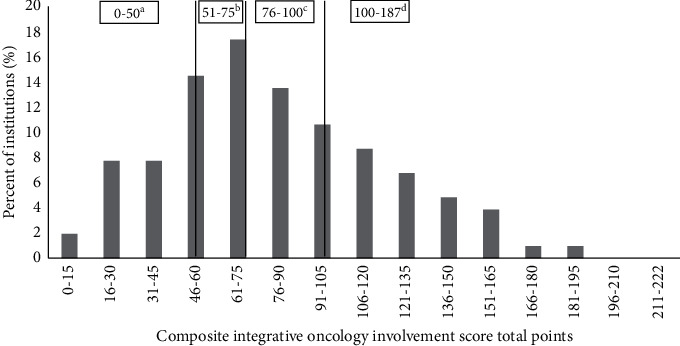
Distribution of 103 institutions by their composite integrative oncology involvement score. (a) The score of 0–50 indicates “low” integrative oncology involvement (*n* = 25). (b) 51–75 indicates the “low-mid” level (*n* = 26). (c) 76–100 indicates “mid-high” level (*n* = 23). (d) 101–187 indicates a “high” level (*n* = 29).

Among the 103 institutions, there was a wide distribution of integrative involvement scores, indicating a wide variety of integrative oncology practices circa 2013–2014 (see Figure 2). Based on this distribution, institutions were divided into four quartiles for integrative involvement, indicated by black lines.

Considering individual components of the composite score, most points were awarded based on “educate” and “support” across the 12 therapies; the levels for “provide” across the 12 were notably lower in all but the high integrative score category.  Table 2 shows the average points awarded from each category across the 12 modalities, cut by the institutions' integrative score.

Using “educate” as a leading indicator, some modalities emerge as more prevalent than others.  Table 3 is aggregated across the four components of the “educate” definition to show how the education levels varied across the integrative score. The therapies with the most efforts made by institutions to raise awareness and knowledge are nutrition consultations (76–100%), exercise consultations (68–100%), patient support groups (80–100%), spiritual services (48–100%), and psycho-oncology support groups (56–97%). Patients were offered some form of awareness-raising for these core modalities in all but the lowest quartile of institutions, being offered in 48–80% of institutions in the low group compared to 97–100% in the high group.

Table 4 illustrates the percentage of patients receiving conventional medical treatment at any point during the 5 years after diagnosis. As the timeframe for diagnosis was January 2013 to December 2014, the use of immuno-oncology agents and targeted therapies was minimal.

The patient mortality marker analysis of the integrative cohorts showed a directional finding (Table 5). Though there is no statistical significance at *p* < 0.05, institutions in the low cohort have a notably lower 5-year overall survival rate (89%) compared to institutions in the low-mid (96%), mid-high (96%), and high (95%) cohorts.

Based on multivariate modelling, older age at diagnosis (66–76), having a PPO insurance plan, being treated in an academic setting, and being treated by an institution with a low-mid or mid-high integrative involvement score were predictors of increased odds of 5-year survival; see Table 6. Several other factors lowered the odds of 5-year survival, including metastatic positive, being treated at a location that is not NCCN designated, being treated in the Midwest or West regions, being aged 55–65 at diagnosis, and being treated at a location that is contracted or employed by an ACO.

An odds ratio of greater than one is expressed directly in relation to 5-year survival. Using the “low-mid” integrative score as an example, a patient treated at an institution with a “low-mid” integrative score is three times more likely to survive over the 5-year period, compared to a patient treated at an institution with a “low” score. On the contrary, an odds ratio of less than one indicates lower survival. Using “metastatic positive” as an example, a patient who is metastatic positive is 50 times more likely to die in the 5-year period compared to a patient who is “metastatic negative.”

## 4. Discussion

Many cancer institutions support a wide array of integrative oncology services, from core supportive services such as nutrition and psycho-oncology to less common services such as acupuncture, massage, and reiki. However, about one-quarter of institutions score low in these types of services. Low-scoring institutions raised awareness of common services less than 80% of the time, whereas all other institutions raised awareness of such services near or over 80% of the time. Low-scoring institutions also did less to provide access and funding to integrative oncology than all other institutions. Compared to low-scoring institutions, the odds of 5-year survival for breast cancer patients treated at institutions with low-mid and mid-high integrative involvement were three times and 48% higher, respectively. The magnitude of this survival advantage is comparable to being treated at an academic medical centre or an NCCN-designated centre.

The study has some limitations which are as follows: the sample is limited to patients filing under commercial insurance; patients may not all be de novo due to a two-year lookback period; oncologist recall from 6–7 years prior may be subject to error; the scoring system is based on the authors' understanding of methods used by institutions to educate, support, and provide access to complementary and lifestyle therapies but has not been validated; the multivariate model is limited to the variables included and could benefit from additional patient characteristics; and conventional medical treatments were shown to be similar across the integrative cohorts, but no adjustments were made to the model to account for treatment variation.

Regarding the role of the integrative score, there are no secondary sources to directly compare the results. The main finding is that patients' survival odds increase at either the low-mid or mid-high level of integrative involvement, but survival odds are neutral at the high level. One possible reason is that patients treated at high-scoring institutions may be correlated with patients who are more severe and/or have had cancer longer and are therefore looking for institutions that offer “more” through whole-person approaches. Additional patient variables would need to be included to test this hypothesis. Validation of the composite score calculation as a representation of involvement in integrative oncology practice and repetition of the experiment are needed.

When going through cancer treatment, patients rely heavily on the information provided to them by their oncologist and the larger healthcare team. Patients cannot use complementary or lifestyle therapies if they are not aware of them, and they may be hesitant to change current practices if these therapies are not supported at their treating institution. Convenience also plays a role; patients may not be willing or able to travel to other locations if additional modalities are not offered at their main location for cancer treatment. Finally, cost is one of the known barriers to more patients' use of integrative approaches. If a minimal threshold of integrative involvement is all that is needed to increase the odds of survival, as shown in this study, then this is important to patients, to institutions, to healthcare professionals that treat them, and to pharmaceutical companies providing conventional treatments. Increasing the odds of survival through whole-person integrative oncology is an endeavour worthy of additional investigation and investment by the treating institutions and pharmaceutical companies. In particular, there are national charitable organizations, such as Unite for HER, that have a proven model to provide education and funding of complementary and lifestyle therapies and represent a path to increasing integrative access for patients. In addition, the Samueli Foundation, the SIO (Society of Integrative Oncology), and ASCO (American Society of Clinical Oncologists) are working to increase the ability of oncologists to discuss and assist patients to learn about and access such services in appropriate ways.

Showing the benefit of adding specific integrative therapies to breast cancer patients is the basis of the SIO guidelines published in 2017 and approved by ASCO [13]. In these guidelines, specific modalities are recommended to address anxiety and mood disorders (meditation, yoga, and relaxation with imagery) or to address stress reduction, anxiety, depression, fatigue, and quality of life (stress management, yoga, massage, music therapy, energy conservation, and meditation). Many of these modalities can be facilitated by trained psychologists, further highlighting the importance of access to psycho-oncology services for cancer patients. Additional joint guidelines from SIO and ASCO for pain and fatigue are in development.

Although 12 modalities were researched in this study, five are highlighted in the results that are more commonly adopted. These are exercise counselling, nutrition counselling, psycho-oncology support, chaplain services, and patient support groups. When checking the SIO guidelines, meditation is listed for both mood disorder management and general stress reduction. We recommend adding meditation as a sixth key “core modality.” These six represent an attractive bundle that addresses patients' needs physically, mentally, socially, and spiritually, is often accepted as part of usual care, and provides some degree of choice for patients. Treating institutions can cross the threshold into low-mid involvement in numerous ways; one specific plan to do so is as follows: (1) educating patients about the core six complementary and lifestyle modalities in print, on the website, and in discussion during a treatment visit. (2) Offering a path to access three on-site (for example, nutrition counselling, exercise counselling, and psycho-oncology support) and the other three at a referred location (patient support groups, chaplain services, and meditation). (3) Covering the costs of the three offered on-site. (4) Including on-site staffing of a nutritionist, exercise consultant/physical therapist, and psychologist. This requires resources and training and should be planned for accordingly.

Finally, this study concludes that institutional support for complementary and lifestyle therapies increases the odds of patient survival, which may initially seem to contradict a 2018 study published in the Journal of the National Cancer Institute. The 2018 study suggests that patients who choose alternative treatments and eschew conventional medical treatments have significantly worse survival than patients who receive conventional treatment only [14]. The difference is in the use of conventional medical treatments as a basis; the 2018 study used *alternative* modalities, while the assumption in this study is that patients are using complementary and lifestyle therapies in *addition* to their medical treatments, supported by “integrative involvement” being derived from actions taken by the institution and the medical team treating the patient. Both studies point to the importance of oncologists to address behavioural, lifestyle, and psychosocial issues with patients as well as alternative modalities to assure that all beneficial services—conventional and complementary—are properly provided in patient care. Proper “integration” of these services can improve both quality of life and survival and must become the standard of care for all cancer patients.

## 5. Conclusion

Institutions crossing the threshold beyond “low” involvement in integrative oncology represent a survival benefit for many cancer patients. Treating institutions should invest in six core complementary and lifestyle therapies and integrate them with conventional medical treatments. Other entities that support patients on their cancer treatment journey—such as pharmaceutical companies and national or regional payers—should also investigate the socioeconomic benefits of providing education and access to these six core therapies. Once these modalities are made more widely available and accessible to all cancer patients, the survival benefit can be realized.

## Figures and Tables

**Table 1:** Survey questions and response points used to develop a composite integrative involvement score

Survey question construct	Question phrasing	Response instructions	Response options	Integrative score points	Maximum points possible
Educate	Efforts by the institution to raise patient awareness and knowledge of 12 integrative modalities	Choose all that apply; asked for each of the 12 modalities	Flyers or other printed information posted in-office or online for patients to see	1	60
			Written or printed information provided to the patient	1	
			Proactive discussion had with the patient (by oncologist or member of cancer team)	2	
			Other methods of raising awareness	1	
			Unsure/not promoted	0	

Support	Efforts taken by the institution to offer or otherwise recommend 12 integrative modalities	Choose one (with the exception of the first two responses, which could both be chosen); asked for each of the 12 modalities	Offered at my primary practice institution (my location)	3	60
			Offered at my primary practice institution (another affiliated location)	2	
			Not offered, but patients are referred to places that offer	1	
			Not offered, but may be recommended	1	
			Unsure/not supported	0	

Provide	Institution in part or full covering the costs of 12 integrative modalities	Choose one; asked for each of the 12 modalities	Yes, costs covered fully	4	48
			Yes, costs covered in part	3	
			No, patient responsible for all costs	0	
			Unsure/did not offer	0	

Staffing	Institution keeping specific roles on staff as part of cancer patient care team	Choose one; asked in isolation	Social worker, oncology patients	3	51
			Patient navigator, oncology patients	3	
			Financial assistant, oncology patients	3	
			Leader or director of integrative services	12	
			Credentialed acupuncturist or acupressurist	6	
			Credentialed massage therapist	6	
			Credentialed nutritionist	6	
			Pain specialist, oncology patients	3	
			Physical therapist, oncology patients	3	
			Psychologist, oncology patients	3	
			Patient liaison	3	

Supplements	Efforts taken by the institution to help patients address the use of herbal and plant-based supplements	Choose one; asked in isolation	Yes, offered at my primary practice institution (main location or satellite locations in-network)	3	3
			No, not offered, but would refer patients to other resources (for example, Memorial Sloan Kettering website)	2	
			No, not offered, but I or my staff would help patients with questions on a case-by-case basis	1	
			No, was not offered	0	
			Unsure	0	

The range of total points possible is 0-222.

**Table 2:** Integrative score from 2013 to 2014, average points across 12 complementary and lifestyle modalities.

Survey construct measured	Maximum points possible	Low integrative score	Low-mid integrative score	Mid-high integrative score	High integrative score
Educate	60	10	17	23	33
Support	60	10	18	22	29
Provide	48	4	8	14	27

**Table 3:** Percentage of institutions educating patients in any way about select complementary and lifestyle therapies circa 2013–2014.

	Low integrative score (*n* = 25) (%)	Low-mid integrative score (*n* = 26) (%)	Mid-high integrative score (*n* = 23) (%)	High integrative score (*n* = 29) (%)
Nutrition consultation or program	76	96	100	100
Exercise consultation or program	68	85	87	100
Patient support groups or patient-survivor pairings	80	96	96	100
Spiritual services	48	85	83	100
Psycho-oncology support	56	92	78	97
Massage therapy	32	69	87	97
Meditation or mindfulness	20	77	91	97
Yoga	24	73	87	97
Acupuncture or acupressure	20	69	78	93
Music or art therapy	24	58	74	93
Reiki or therapeutic/healing touch	16	35	48	72
Tai chi or qi gong	12	31	44	72

**Table 4:** Percentage of patients receiving conventional medical treatments in any line of therapy postdiagnosis, circa 2013–2014.

Conventional medical treatment	Low integrative score (*n* = 35) (%)	Low-mid integrative score (*n* = 48) (%)	Mid-high integrative score (*n* = 26) (%)	High integrative score (*n* = 64) (%)
Chemotherapy (monotherapy)	43	50	42	27
Chemotherapy + immuno-oncology agent	9	17	12	9
Chemotherapy + targeted therapy	0	0	0	2
Immuno-oncology agent (monotherapy)	3	4	4	0
Targeted therapy (monotherapy)	0	2	0	5
Hormone therapy (monotherapy or in combination)	51	67	88	73
Surgery (excluding diagnostic procedures)	91	98	85	94
Radiation	69	56	69	48

**Table 5:** 5-year survival across varying levels of institutional integrative involvement.

Institution integrative oncology score	Oncologist sample	Patient sample	Survival rate (%)
Low	25	35	89
Low-mid	26	48	96
Mid-high	23	26	96
High	29	64	95
Total	103	173	94

**Table 6:** Lasso regression of 5-year survival: variable coefficients and odds ratios.

Variables	Coefficient	Odds ratio	Variable comparison
Metastatic positive	−3.99	0.02	Relative to metastatic negative
Not NCCN designated	−1.74	0.18	Relative to NCCN designated
Midwest region	−1.70	0.18	Relative to Northeast region
West region	−1.60	0.20	Relative to Northeast region
Age 56–65	−0.74	0.48	Relative to age 32–45
ACO contracted or employed	−0.08	0.92	Relative to no ACO relationship
High integrative score	0	1.00	Neutral
South region	0	1.00	
Age 46–55	0	1.00	
Not NCI designated	0	1.00	
Mid-high integrative score	0.39	1.48	Relative to low score
Academic setting	0.96	2.61	Relative to community setting
Low-mid integrative score	1.13	3.10	Relative to low score
PPO patient insurance	1.20	3.32	Relative to “other insurance” (HMO/indemnity/POS)
Age 66–76	1.36	3.90	Relative to age 32–45

NCCN, National Comprehensive Cancer Network; ACO, Accountable Care Organization; NCI, National Cancer Institute; PPO, preferred provider organization; HMO, health maintenance organization; POS, point of service.

## Data Availability

The data underlying this article that is HIPAA-compliant will be shared on reasonable request to the corresponding author.
